# Calcium Regulation of EGF-Induced ERK5 Activation: Role of Lad1-MEKK2 Interaction

**DOI:** 10.1371/journal.pone.0012627

**Published:** 2010-09-07

**Authors:** Zhong Yao, Seunghee Yoon, Eyal Kalie, Ziv Raviv, Rony Seger

**Affiliations:** Department of Biological Regulation, Weizmann Institute of Science, Rehovot, Israel; Louisiana State University, United States of America

## Abstract

The ERK5 cascade is a MAPK pathway that transmits both mitogenic and stress signals, yet its mechanism of activation is not fully understood. Using intracellular calcium modifiers, we found that ERK5 activation by EGF is inhibited both by the depletion and elevation of intracellular calcium levels. This calcium effect was found to occur upstream of MEKK2, which is the MAP3K of the ERK5 cascade. Co-immunoprecipitation revealed that EGF increases MEKK2 binding to the adaptor protein Lad1, and this interaction was reduced by the intracellular calcium modifiers, indicating that a proper calcium concentration is required for the interactions and transmission of EGF signals to ERK5. In vitro binding assays revealed that the proper calcium concentration is required for a direct binding of MEKK2 to Lad1. The binding of these proteins is not affected by c-Src-mediated phosphorylation on Lad1, but slightly affects the Tyr phosphorylation of MEKK2, suggesting that the interaction with Lad1 is necessary for full Tyr phosphorylation of MEKK2. In addition, we found that changes in calcium levels affect the EGF-induced nuclear translocation of MEKK2 and thereby its effect on the nuclear ERK5 activity. Taken together, these findings suggest that calcium is required for EGF-induced ERK5 activation, and this effect is probably mediated by securing proper interaction of MEKK2 with the upstream adaptor protein Lad1.

## Introduction

The extracellular signal-regulated kinase5 (ERK5) signaling cascade is composed of MEKK2/3 at the MAP3K tier [1], [2], MEK5 at the MAPKK tier [3] and ERK5 itself as the mitogen-activated protein kinase (MAPK) component [3], [4]. This cascade was initially thought to respond to stress stimuli only, but was later shown to be essential also for mitogenesis [5], which could be mediated by its role in cell cycle progression [6], [7], and together with ERK2, in oncogenic transformation [8], [9]. In addition, the ERK5 cascade plays a role in the regulation of differentiation [10], [11], migration [12], [13], [14], neuronal survival [15], embryonic angiogenesis [16], serial assembly of sarcomeres [17], determination of cortical neuronal fate [18] and more [19], [20].

The molecular mechanism of activation of the ERK5 cascade is not fully elucidated yet. It seems that the cascade can be activated by more than one mechanism dependent on the extracellular stimulus. Thus, the protein tyrosine kinase c-Src [21], the small GTPase Ras [22], the adaptor protein Lad1 [23] and the protein Ser/Thr kinase WNK1 [24] were all implicated in the activation of the cascade under various conditions. However, the conditions under which the different components operate and the inter-relationships between them still need clarification. Upon activation of MEKK2/3 by any one of the mechanisms, these MAP3Ks interact with MEK5 via specific PB1 domains [25], [26]. This interaction then allows phosphorylation of MEK5 on Thr and Ser residues in its activation loop, which consequently induces the activation of this MAPKK component. MEK5 is specific towards ERK5, phosphorylating it on its activation loop Tyr and Thr residues, activating this MAPK, and thus defines the pathway as a distinct MAPK cascade [3]. A possible alternative route for ERK5 activation acts via Gq protein-coupled receptor signaling, in which Gαq acts as a scaffold protein to recruit PKCξ that phosphorylates and activates MEK5 [27]. Finally, at the MAPK level, the 110 kDa ERK5 seems to serve as the only active component. Its N-terminal part shares about 50% identity with ERK1/2, while its unique C-terminal part, which is 400 amino acids long, has no similarity to any known kinase [19], [20].

As other MAPK cascades, the ERK5 cascade seems to function mainly through regulation of transcription. ERK5 was shown to directly phosphorylate, and thereby activate, members of the MEF2 group of transcription factors, including MEF2C, which consequently induces the transactivation of the genes like c-Jun [28] and MEF2 [29]. ERK5 also activates other transcription factors such as c-Myc [30], c-Fos [22], [31], Fra-1 [32], SAP1a, [22], peroxisome proliferator-activated receptor delta (PPARδ), [33], and probably also PPARγ [34] and NFκB [9]. Importantly, ERK5 was shown to posses an intrinsic transcriptional activity, which was demonstrated to induce the Nur77 gene transcription upon calcium signals in T cells [35]. This activity is mediated by the non-phosphorylating C-terminal part of the ERK5, and seems to be dependent on heavy autophosphorylation of this region of the kinase [36].

Here we studied the role of calcium, a critical and highly versatile second messenger [37], [38] that was previously shown to modulate the activity of the ERK1/2 [39], [40], [41] as well as JNK and p38 [42] cascades. Other studies also indicated that the ERK5 cascade is influenced by elevated calcium levels upon induction of cellular stresses like H_2_O_2_ and fluid shear stress [43], [44]. However, essentially no data exist on the role of calcium in mitogenic stimulation of ERK5, and the mechanisms involved in this process. We show here that ERK5 activation by EGF is dependent on proper intracellular calcium levels. We found that this effect is probably mediated through interaction of MEKK2 with the adaptor protein Lad1, but not by other potential activators such as CaMKII, c-Src, or WNK1. These results indicate that similarly to ERK1/2, calcium can regulate the activity of ERK5 by modifying protein interactions of components of the cascade.

## Materials and Methods

### Reagents and antibodies

EGF, BAPTA-AM, ionomycin, PP2, KN-62 and KN-93 were purchased from Sigma (St. Louis, MO, USA). Anti general ERK5, MEK5, HA, tubulin and GFP antibodies (Abs) were purchased from Sigma Israel (Rehovot, Israel). Anti phospho-ERK5 Ab was purchased from BioSource (Camarillo, CA, USA). Anti MEKK2, phospho-tyrosine (pY99) and Lad1 were purchased from Santa Cruz Biotechnology (Santa Cruz, CA, USA). Anti phospho-CREB Ab was purchased from Cell Signaling Technology (Danvers, MA, USA).

### Buffers

Buffer A consists of 50 mM β-glycerophosphate (pH 7.3), 1.5 mM EGTA, 1 mM EDTA, 1 mM DTT and 0.1 mM sodium orthovanadate. Buffer H is Buffer A supplemented with 1 mM benzamidine, 10 µg/ml aprotinin, 10 µg/ml leupeptin and, 2 µg/ml pepstatin-A. Radioimmune precipitation assay (RIPA) buffer consists of 137 mM NaCl, 20 mM Tris (pH 7.4), 10% glycerol, 1% Triton X-100, 0.5% deoxycholate, 0.1% SDS, 2 mM EDTA, 1 mM PMSF, 1 mM sodium orthovanadate and 20 µM leupeptin. Kinase reaction buffer (x3) consisted of 75 mM β-glycerophosphate (pH 7.3), 30 mM MgCl_2_, 1.5 mM DTT, 0.15 mM sodium orthovanadate, 3.75 mM EGTA, 0.3 mM ATP and 30 µM calmidazolium.

### Plasmids and constructs

rMEK5α-1 was a gift from Dr. J.D. Lee (Scripps Institute). MEK5-GFP was created by subcloning into pEGFP-N1 between HindIII and SacII sites. MEK5(K/A) was generated by replacing Lys194 to Ala. HA-ERK5 was a gift from Dr. J.S. Gutkind (Oral and Pharyngeal Cancer Branch, NIH). ERK5(1–397)-GST fusion protein was generated by inserting into BamHI and XhoI sites in pGEX-N1(T4). WNK1 was a gift from Dr. M.H. Cobb (University of Texas Southwestern Medical Center). GFP-WNK1 was created by inserting into HindIII and BamH1 sites of the pEGFP-N1. GFP-Lad1 was prepared by inserting Lad1 into pEGFPC1 vector. GST-Lad1 was created by inserting into EcoRI and SalI sites of pGEX-4T. pMEF2FLuc was a gift from Dr. Ron Prywes (Columbia University). Lad1 shRNA was prepared in pSUPER targeting sequence TGCAGGACTTTCCCTGAGG.

### Cell culture and transfection

HeLa cells were cultured in Dulbecco's modified Eagle's medium supplemented with 10% fetal bovine serum (FBS). Sub-confluent cells were subjected to serum starvation (0.1% FBS) for 18 hours prior to stimulation. For transient overexpression, HeLa cells were transiently transfected with the different plasmids using Polyethyleneimine (PEI) [45].

### Protein extraction and immunoprecipitation

The cells were washed twice with phosphate buffered saline and once with Buffer A, scraped into Buffer H, and disrupted by sonication. For immunoprecipitation, the cell extracts were incubated with the proper Abs conjugated to protein A/G beads at 4°C for 2 h. The beads were washed once with RIPA buffer and twice with Buffer A. The immunoprecipitated complexes were then boiled in sample buffer and subjected to Western blot analysis using the indicated Ab. For coimmunoprecipitation, the beads were washed three times with buffer containing 20 mM HEPEs (pH 7.4), 2 mM MgCl_2_, 2 mM EGTA, 150 mM NaCl and 0.1% Triton X-100.

### Western blot analysis and quantification

Samples were subjected to 8 or 10% SDS-PAGE and transferred to nitrocellulose membranes. Membranes were blocked with 2% bovine serum albumin (BSA) in TBS-T (200 mM Tris-HCl pH 8.0, 150 mM NaCl, and 0.05% Tween-20), and then incubated with the appropriate Abs. Membranes were developed with alkaline phosphatase (AP) or horseradish peroxidase (HRP)-conjugated anti mouse or anti rabbit Abs, followed by detection with BCIP/NBT color development substrate (Promega, Madison, WI) or enhanced chemiluminescence (ECL) reagents (Amersham, Arlington Heights, IL), respectively. The intensity of the developed bands was well within the linear range of detection. The blots were scanned and the density of each band was analyzed with ImageJ. Each experiment was repeated at least three times and T-test was used to find statistic significance.

### *In vitro* phosphorylation assay

HeLa cells were transfected with MEK5-HA and were serum-starved for 18 hours. After different treatments, MEK5-HA was immunoprecipitated using anti HA Ab. MEK5 activity was determined by phosphorylating K/A-ERK5(1–397)-GST (2 mg/ml) in kinase reaction buffer with [γ^32^P]-ATP (30°C, 20 min). For MEKK2 activity, endogenous MEKK2 was immunoprecipitated using anti MEKK2 Ab. MEKK2 activity was determined by the phosphorylating MEK5-K/A-GST.

### Luciferase assay

HeLa cells were grown in 24-well plates and transfected with pMEF2FLuc and pRL-TK with PEI. The cells were serum starved for 16 hrs. Three hours after the different treatments, luciferase activities were measured using Dual-Luciferase Reporter kit (Promega, Madison, WI) according to the product manual.

### *In vitro* binding assay

MEKK2 was immunoprecipitated from cell lysates and incubated with 2 µg GST-Lad1 for overnight in a buffer containing 50 mM β-glycerophosphate, 100 mM NaCl, 1 mM DTT supplemented with various concentrations of calcium. Each sample was washed four times with the buffer containing the same level of calcium.

### Immunostaining

HeLa cells were plated on glass cover slips under standard culture conditions. After serum-starvation, the cells were treated with different stimuli and inhibitors as indicated, and the treatment was terminated by 3 washes with PBS. Then, the cells were subjected to fixation (3% PFA, 20 min), permeabilization (0.2% Triton X-100, 4 min), and sequential incubation with the appropriate primary Abs (1∶100, 45 min each) and secondary Abs (1∶100). The cells were visualized by fluorescence microscopy at 100X magnification.

## Results

### Intracellular calcium levels affect ERK5 phosphorylation

**Figure 1:**
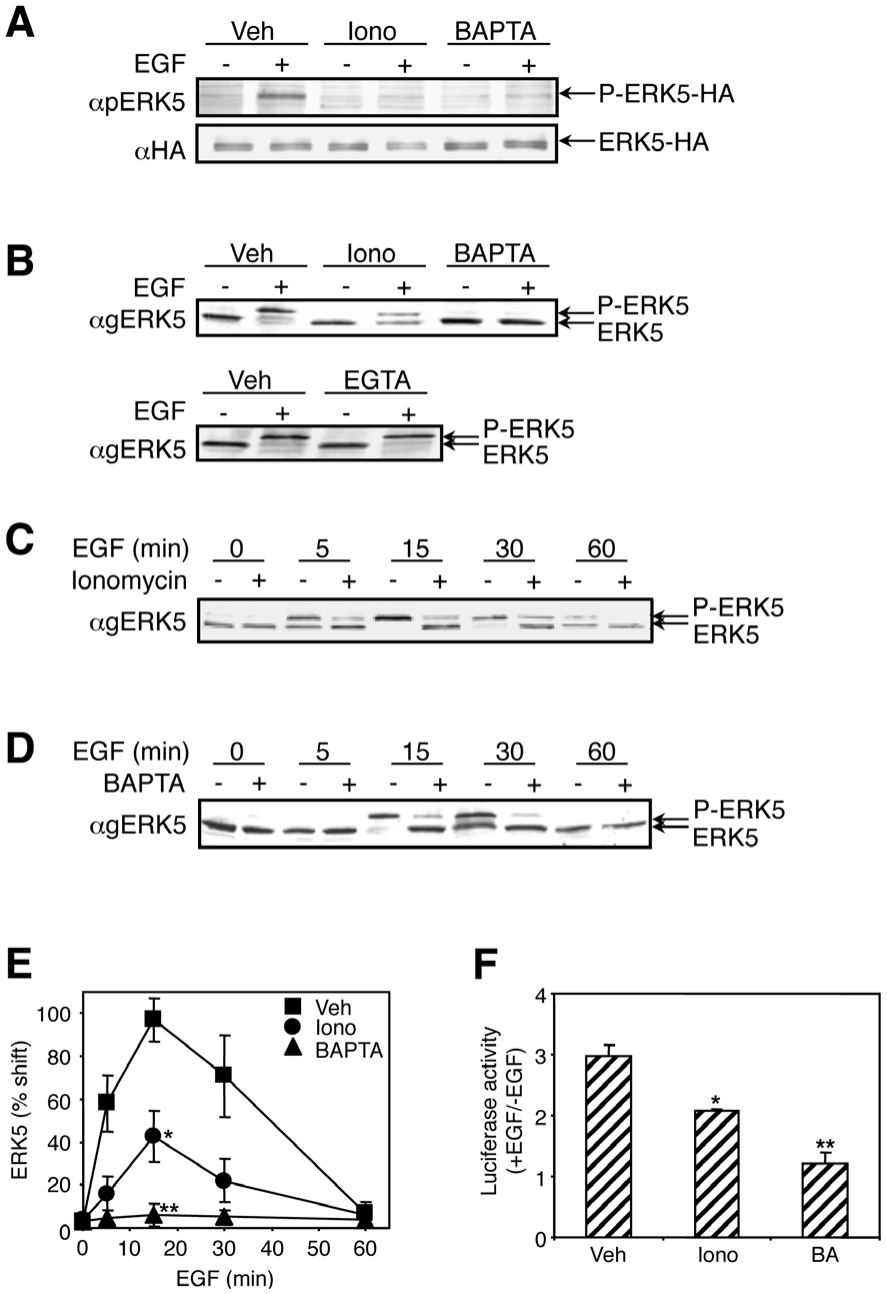
Intracellular calcium levels affect ERK5 activation. (**A**) ERK5-HA was transfected into HeLa cells. After starvation, the cells were treated with vehicle, ionomycin (1 µM) or BAPTA-AM (15 µM) for 15 min and then with EGF (20 ng/ml) for 15 min. ERK5 phosphorylation was detected with anti pERK5 Ab. The experiment was reproduced five times. (**B**) Activation of endogenous ERK5 from cells after different treatments was detected by up-shift of ERK5 detected by anti general ERK5 Ab. The experiment was reproduced five times. (**C–E**) The effects of ionomycin or BAPTA-AM were observed at different time points. Quantification of three independent experiments is presented in (E). The error bars represent standard deviation. P values (*: P<0.05; **: P<0.01) were obtained using T-test by comparing the vehicle control group and the iononmycin- or the BAPTA-AM-treated group. (**F**) HeLa cells were transfected with pMEF2FLuc and pRL-TK. Luciferase activities were measured after different treatments. The value of Y-axis represents the luciferase activity of EGF stimulated sample divided by that without treatment within each pair. The error bars stand for standard deviation of three experiments. P values (*: P<0.05; **: P<0.01) were obtained using T-test by comparing the vehicle control group and the iononmycin- or the BAPTA-AM-treated group.

Calcium, as a major second messenger, influences the ERK1/2 cascade by inducing its activation [39] or, as we recently showed, by modifying protein-protein interactions and subcellular localizations of the ERK1/2 proteins [39], [40]. Interestingly, in a screen aimed to identify upstream regulator of the related ERK5 cascade we found that reduction of cellular calcium concentration resulted in a strong effect on the phosphorylation of ERK5. Therefore, we undertook to study the mechanism by which the ERK5 cascade is regulated by calcium, starting by characterizing the effects of calcium modifiers on ERK5 phosphorylation. For this purpose, we co-transfected MEK5 and ERK5 into HeLa cells, which were then pretreated with either ionomycin, an ionophore that increases the cytosolic calcium concentration, BAPTA-AM, an intracellular chelator of calcium, or EGTA, an extracellular chelator of calcium. Using anti pERK5 Ab, we found that as expected [5], EGF treatment for 15 min significantly induced the activatory double phosphorylation of ERK5 (Fig. 1A). Pretreatment of the cells either with ionomycin or BAPTA-AM prior to EGF stimulation had no effect on the basal activity of ERK5, but resulted in a significant inhibition of exogenous ERK5 phosphorylation. We then examined whether endogenous ERK5 is affected by modulated calcium concentrations as much as the exogenous protein. Since we found that slower migration (up-shifting) of ERK5 in SDS-PAGE is well correlated to ERK5 activation and is much more sensitive than that detected by anti pERK5 Ab, a phenomenon that was also reported in other studies [21], [27], [28], we decided to assess the activation of endogenous ERK5 by its gel shifting. In similarity to the overexpressed protein, also the activation of endogenous ERK5 by EGF was inhibited by modulated calcium concentrations (Fig. 1B). It should be noted that the inhibitory effect of ionomycin on ERK5 activation was slightly more significant in the exogenous ERK5 compared to the endogenous protein (80% vs 60%). The reason for this small difference is not known, but could be derived from improper localization of the overexpressed protein to organelles of the internal calcium stores, (i.e. ER or mitochondria). Upon ionomycin treatment, the calcium levels in these organelles are dramatically reduced [46], the portion of improperly localized ERK5 there is further inhibited, and this increases the inhibition levels of this MAPK.

### Characterization of the calcium effect on ERK5 and downstream transcription

We then further characterized the calcium effects, and found that changes in extracellular calcium levels following EGTA treatment had no effect, nor did it enhance the effect of BAPTA-AM when both were applied to the cells (Fig. 1B and data not shown). These calcium effects were specific to ERK5, as ERK1/2 responded with a stronger basal activation following ionomycin treatment, and a minor augmentation of EGF-induced activity following BAPTA-AM treatment (data not shown). Importantly, these effects were not confined to HeLa cells, as the response of ERK5 to calcium in Rat1 cells was very similar (data not shown). We then followed the effects of BAPTA-AM and ionomycin on ERK5 over time (Fig. 1C–E) and found that while BAPTA-AM completely inhibited ERK5 phosphorylation, ionomycin treatment resulted only in partial inhibition of the signal, and these effects were consistent throughout the treatment.

We further examined whether the effects of modulated calcium levels were transmitted to targets of ERK5, and MEF2 was selected for this purpose since it is a well-studied substrate of ERK5 [47]. Thus, pMEF2FLuc, a construct containing luciferase downstream to a promoter with MEF2 binding sites, was introduced into HeLa cells. After stimulation with EGF alone, we observed significant increase of luciferase activity (Fig. 1F). When the cells were pre-treated with BAPTA-AM, the EGF-induced increase of activity was abolished. On the other hand, treatment with ionomycin increased the basal luciferase activity. This may be explained by the fact that other calcium signaling components such as calcineurin facilitate the activation of MEF2s [48] under these conditions. However, the fold increase of luciferase activity induced by EGF stimulation was significantly lower than that with vehicle control. This result is consistent with the results obtained with ERK5 phosphorylation, confirming that calcium levels influence ERK5 activity.

### Calcium does not significantly affect MEK5 activity and interactions

**Figure 2:**
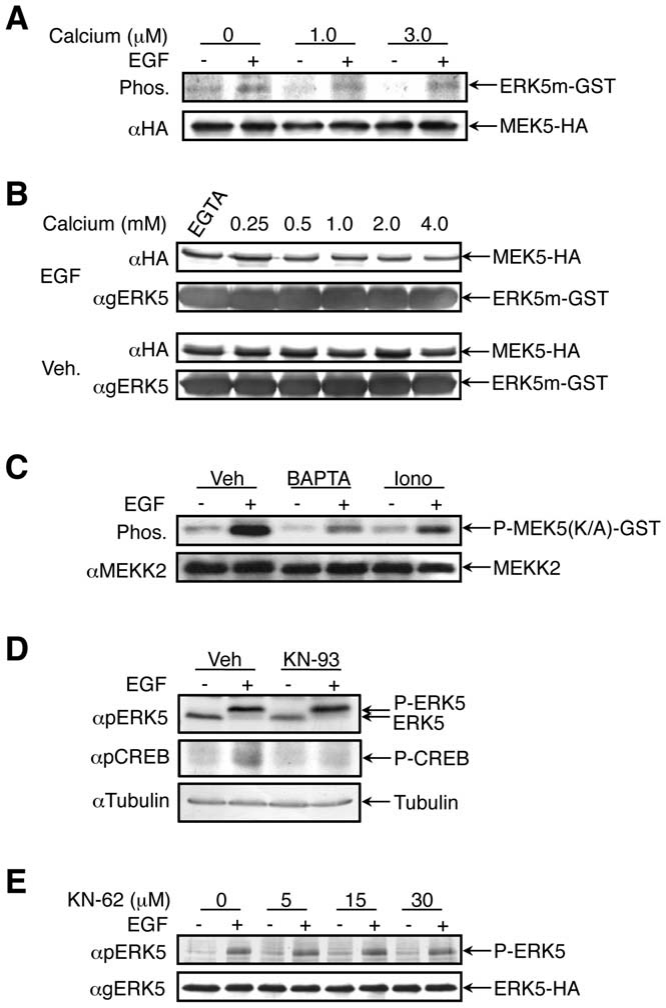
Calcium modifiers affect either MEKK2 or its upstream components, without involvement of CaMKII. (**A**) MEK5-HA transfected HeLa cells were either stimulated with EGF or left untreated. MEK5-HA was immunoprecipitated and its in vitro kinase activity towards K/A ERK5 (1**–**397)-GST was measured in the presence of the indicated calcium concentrations. (**B**) Exogenously expressed MEK5-HA was immunoprecipitated from HeLa cells, which were either stimulated with EGF (20 ng/ml, two upper panels) or were left without treatment (two lower panels). The MEK5 was than subjected to in vitro binding assay with K/A ERK5(1**–**397)-GST, in the presence of indicated calcium concentrations or calcium chelator EGTA. (**C**) MEKK2 was immunoprecipitated from the cells treated with vehicle, BAPTA-AM or ionomycin in combination with or without EGF (20 ng/ml). Its activity was measured by the incorporation of radioactive phosphate to recombinant MEK5-GST. (**D**) HeLa cells were pretreated with 30 µM of KN-93 for 30 min and then stimulated with EGF (20 ng/ml) for 10 min. ERK5 activation was measured by band up-shift of endogenous ERK5 detected by anti general ERK5 Ab, CREB phosphorylation was detected by anti pCREB Ab, and anti tubulin Ab was used as a loading control. (**E**) ERK5-HA transfected HeLa cells were pretreated with KN-62 in different concentrations for 1 h. After EGF stimulation, ERK5-HA activation was measured with anti pERK5 Ab in Western blot analysis. All the experiments in this figure were reproduced at least three times.

The activity of a number of kinases was shown to be affected directly upon changes in calcium levels, as was well established for the family of Ca^++^/Calmodulin-dependent kinases (CaMK). We therefore undertook to examine the possible role of calcium as a cofactor that directly influences MEK5 activity towards ERK5. For this purpose, the phosphorylation of K/A-ERK5(1-397)-GST by MEK5 was assessed in vitro with different calcium concentrations in the reaction solution. We used the ATP binding site mutant which is catalytically inactive in order to eliminate the possible effect of feedback phosphorylation by ERK5 on its interaction However, 1 µM or 3 µM calcium in the reaction had essentially no effect on the activity of MEK5 towards ERK5 in vitro (Fig. 2A), indicating that the examined concentrations of calcium do not affect MEK5 activity.

Another possible mechanism for inhibition of ERK5 phosphorylation is a direct interference with MEK5-ERK5 interactions. To address this possibility, we used again the inactive isoform of ERK5, (K/A-ERK5(1**–**397)-GST). This construct was attached to glutathione beads and used for a GST-pulldown assay. Extracts of MEK5-HA transfected HeLa cells were then incubated with the beads in the absence of calcium (EGTA) or in the presence of different calcium concentrations. Following washing of the beads, levels of specifically bound MEK5-HA were assessed by Western blot. No effect on the interaction was detected when µM calcium concentrations were used, although higher (mM) concentrations slightly reduced the binding of MEK5 from both stimulated and unstimulated cells (Fig. 2B). The binding was specific since GST by itself failed to bind MEK5 under the same conditions (data not shown). Since the concentrations here are much above the physiological concentration (0.1 µM in resting cells and up to 1 µM upon stimulation), it is unlikely that calcium modulates the activity or interactions of MEK5.

### The effects of calcium on the ERK5 cascade is upstream of MEKK2 but is not regulated by CaMKII

Next, we undertook to examine whether the inhibition of ERK5 phosphorylation by BAPTA-AM and ionomycin occurs at the level of MEKK2. For this purpose we immunoprecipitated endogenous MEKK2 from treated HeLa cells and examined the activity of the precipitated proteins using in vitro phosphorylation with bacterially expressed MEK5(K/A)-GST (or GST control, data not shown) as a substrate. Indeed, MEKK2 from EGF-stimulated cells phosphorylated MEK5 much stronger than the phosphorylation by the protein precipitated from non-stimulated cells (Fig. 2C). These results are not affected by autophosphorylation of the recombinant MEK5 since the K/A mutation renders this kinase inactive. We further examined the effects of BAPTA-AM and ionomycin by repeating the procedure with HeLa cells pretreated with these compounds. We found that BAPTA-AM completely inhibited MEKK2 activity towards MEK5, whereas ionomycin caused a weaker inhibition, corresponding to the endogenous inhibition of the ERK5 signal (Fig. 1). These experiments suggest that calcium affects the ERK5 cascade either at the MAP3K or at a further upstream level. However, since it is unlikely that calcium remains bound to the immunoprecipitated MEKK2, we favor the possibility that the calcium interferes with upstream steps in the MEKK2-ERK5 activation. CaMKII is a major effector in calcium signaling, with a broad spectrum of substrates. It has been previously shown that activation of the ERK1/2 cascade may be dependent upon CaMKII in neurons [49], and that this dependence underlies ERK1/2 activation following ionomycin treatment. We therefore undertook to examine the possible role of CaMKII in EGF-mediated ERK5 signaling. For this purpose, we pretreated HeLa cells with KN-93, a specific CaMKII inhibitor [50], and then stimulated with EGF (Fig. 2D). We found that although the inhibitor efficiently reduced the EGF-induced phosphorylation of CREB, which is one of the CaMKII substrates [51], it failed to inhibit ERK5 phosphorylation. The lack of CaMKII effect was confirmed by another CaMKII inhibitor, KN-62 [52], which again had no significant effect on the EGF-induced ERK5 activation under any concentration used (Fig. 2E).

### Lad1 is involved in ERK5 activation by EGF

**Figure 3:**
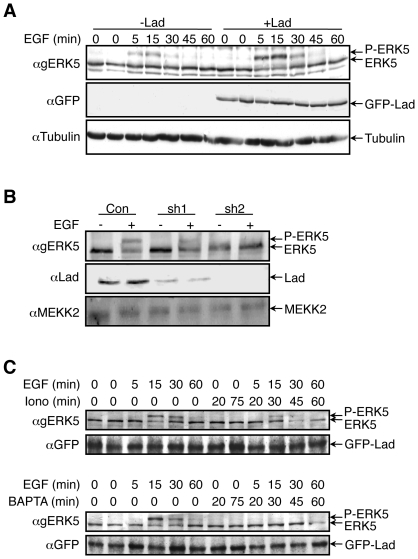
EGF-induced ERK5 phosphorylation is mediated by c-Src and Lad1 but not by WNK1. (**A**) HeLa cells were transfected with GFP-Lad1 or vector control followed by treatment with EGF (20 ng/ml) for the indicated times. ERK5 phosphorylation was determined using upshift of ERK5 in blot. (**B**) Lad1 shRNA (Sh1**–**2 µg or Sh2**–**6 µg) or empty vector (con) were transfected into HeLa cells. The cells were stimulated by EGF and ERK5 phosphorylation was determined by Western blot as in (A). The levels of endogenous Lad1 and MEKK2 were determined by their corresponding Abs as indicated. (**C**) HeLa cells were transfected with GFP-Lad1 and the activation of ERK5 in response to EGF in the presence of ionomycin or BAPTA-AM was detected by band shift. All the experiments in this figure were reproduced at least three times.

Lad1 was identified as an adaptor protein that associates with MEKK2, and thereby regulates the ERK5 activation by EGF [25]. Since the effects of calcium on ERK5 activity were mediated by processes upstream of MEKK2, we undertook to examine the role of the Lad1 in this process. First we verified that Lad1 is indeed involved in the EGF-ERK5 pathway by modulating the expression of Lad1 in HeLa cells. Indeed, when GFP-Lad1 (1**–**366) was overexpressed in the cells, ERK5 phosphorylation upon EGF treatment was much higher than in control transfected cells (4+/−0.8 fold, Fig. 3A) without any significant change in the duration of the signal. On the other hand, reduction in Lad1 expression by a specific shRNA resulted in a significantly lower ERK5 phosphorylation upon EGF treatment (Fig. 3B). We did not observe the reported participation of WNK1 in EGF-induced stimulation of ERK5 in HeLa cells [24], as in our system overexpression of WNK1 actually reduced the EGF-stimulated ERK5 gel retardation (data not shown). Together, these data indicate that Lad1, but not WNK1, is likely to be involved in EGF-induced ERK5 activation in our system.

We then examined whether Lad1 may influence the effects of modified calcium levels on EGF-induced ERK5 activation. For this purpose, GFP-Lad1 was overexpressed in HeLa cells, which were later serum-starved and stimulated with EGF in the presence or absence of BAPTA-AM and ionomycin. Similar to non-transfected cells (Fig. 1), pretreatment of the Lad1-overexpressing cells with ionomycin inhibited this activation for about 60% (Fig. 3C upper panels) and BAPTA-AM abolished the EGF-stimulated ERK5 in these cells (Fig. 3C lower panels). Therefore, Lad1 does not protect or modify the ERK5 cascade from the effects of changing calcium concentrations, indicating that these effects are not likely to occur upstream of this component. Since we ruled out an influence of calcium downstream of Lad1-MEKK2, the interaction of Lad1 with MEKK2 might be the responsive step to changes in calcium concentrations.

### EGF induces a calcium-dependent MEKK2-Lad1 interaction

**Figure 4:**
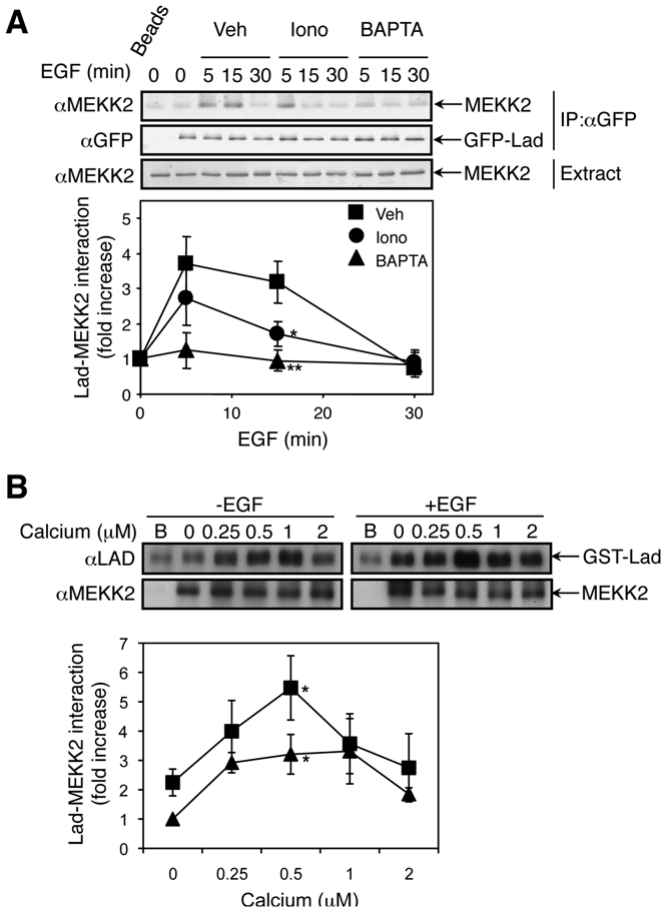
MEKK2-Lad1 interaction is modulated by calcium. (**A**) Exogenously expressed GFP-Lad1 was immunoprecipitated from HeLa cells with indicated treatments. The MEKK2 molecules associated with GFP-Lad1 was detected by anti MEKK2 Ab. The lower panel shows the average quantification of three independent experiments. The error bars represent standard deviation. P values (*: P<0.05; **: P<0.01) were obtained using T-test by comparing the vehicle control group and the iononmycin- or the BAPTA-AM-treated group. (**B**) MEKK2 was immunoprecipitated from HeLa cells without or with treatment of EGF. It was then incubated with recombinant GST-Lad1 in the presence of various concentrations of calcium. The associated GST-Lad1 was detected by anti Lad1 Ab. The lower panel shows the average quantification of three independent experiments. The error bars represent standard deviation. P values (*: P<0.05) were obtained using T-test within each group, EGF-treated or nontreated, by comparing the interaction at 0.25 µM calcium and that without calcium.

In order to study the possible effects of calcium on the interaction of Lad1 with MEKK2, we determined their degree of association using coimmunoprecipiatation. For this purpose GFP-Lad1 was transfected into HeLa cells, and an anti GFP Ab was used to co-immunoprecipitate endogenous MEKK2 with the exogenously expressed GFP-Lad1. We found, as expected [23], that MEKK2 and Lad1 interact with each other in resting cells (Fig. 4A). It has been previously suggested that treatment with EGF may disturb the interaction between the two proteins [23]. However, EGF treatment of the Lad1-transfected cells increased the MEKK2-Lad1 interaction that peaked 5 min after stimulation and was reduced to below basal levels 25 min later. As the previous report showed that the reduced interaction occurs at longer time after stimulation, we concluded that EGF initially induces an increased interaction between the two proteins, and this is reduced to below basal levels only at later time points. Therefore, elevated interaction between MEKK2 and Lad1 is probably required for the transmission of EGF signals towards ERK5.

We then undertook to study the influence of modulated calcium concentrations on the MEKK2-Lad1 interaction. As described above, we pretreated the GFP-Lad1 transfected cells with BAPTA-AM and ionomycin and then studied the degree of interaction upon EGF stimulation. We found that ionomycin partially reduced the EGF-stimulated MEKK2-Lad1 interaction and its effect was maximal (70%+/−12%) 15 min after stimulation. On the other hand, BAPTA-AM abolished the interaction between the two proteins almost completely. These results are in a good correlation with the effect of calcium modulation on MEKK2-ERK5 phosphorylation (Figs. 1 **–** 2), indicating that this effect on ERK5 may be derived from the effects of calcium on MEKK2-Lad1 interaction, which thus becomes a central regulatory process in regulating the ERK5 cascade.

In order to determine whether the calcium effect on MEKK2-Lad1 interaction is direct or mediated by other interacting proteins, we further examined the interaction by an in vitro binding assay. Thus, MEKK2 from HeLa cells treated with or without EGF was immunoprecipitated. After thorough wash, the precipitant was incubated with recombinant GST-Lad1 in the presence of various levels of calcium, and this was followed by three washes with buffers containing the same examined calcium concentrations. Overall, the interaction of Lad1 with MEKK2 was stronger when MEKK2 was immunoprecipitated from EGF-stimulated cells (Fig. 4B). More importantly, addition of calcium indeed modified the MEKK2-Lad1 interaction in both non-treated and EGF-stimulated samples in a biphasic manner. Thus, the binding between the two molecules was weak without calcium or at low calcium concentration. The interaction was elevated in calcium concentrations of 0.5**–**1 µM, which falls within the upper part of stimulated physiological levels. Above 1 µM, the binding was reduced to a comparable level to that of no calcium. Therefore, our results confirm that a narrow range of calcium concentrations is required for strong Lad1/MEKK2 interaction.

### Effect of calcium on the tyrosine phosphorylation of Lad1 and MEKK2

**Figure 5:**
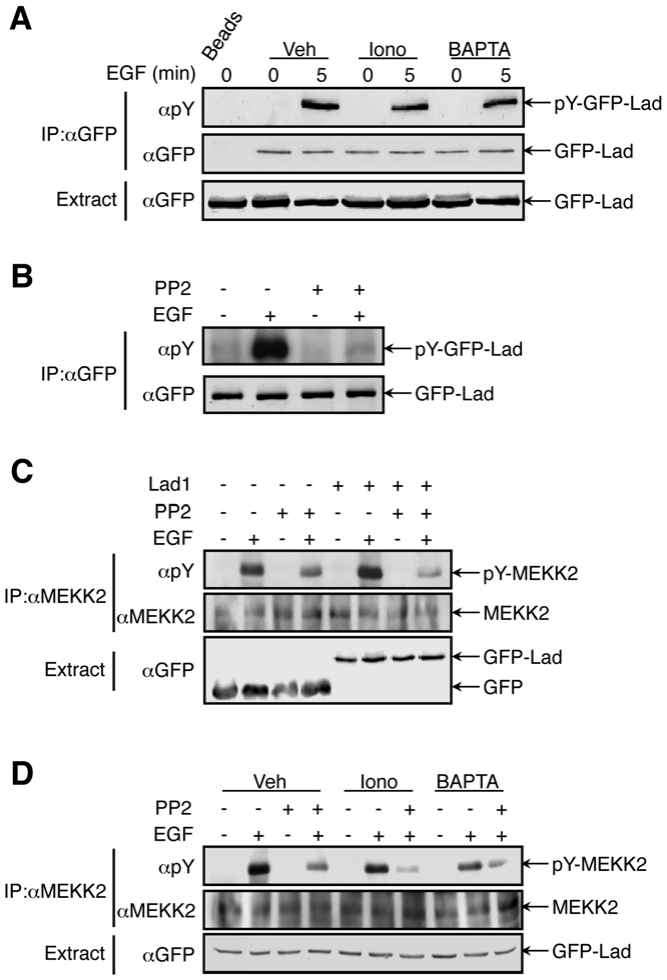
Calcium modulates Tyr phosphorylation of MEKK2 but not of Lad1. HeLa cells transfected with GFP-Lad1 were subject to different treatments as indicated (EGF 20 ng/ml; ionomycin (1 µM) or BAPTA-AM (15 µM) for 15 min; PP2 3 µM). GFP-Lad1 was immunoprecipitated with anti GFP Ab (**A, B**). Endogenous MEKK2 was immunoprecipitated by anti-MEKK2 Ab (**C, D**). Their phsophorylation on Tyr residues was detected by pY99 Ab. The experiments in this figure were reproduced 3 times.

It was previously demonstrated that both Lad1 [53] and MEKK2 [23] are phosphorylated on tyrosine residues upon stimulation. These phosphorylations are likely to be mediated directly by Src family kinases, and were shown to be important for their signaling initiated by these proteins. We therefore assessed the possibility that calcium affects Lad1-MEKK2 interactions by interfering with these phosphorylation events. To study the effect of calcium on the EGF-induced Tyr phosphorylation of Lad1, GFP-Lad1 transfected HeLa cells were pretreated with ionomycin or BAPTA-AM prior to EGF stimulation, and GFP-Lad1 was immunoprecipitated and subjected to Western blot analysis with anti pTyr Ab. As expected GFP-Lad1 was Tyr phosphorylated upon EGF treatment (Fig. 5A), and this phosphorylation was slightly reduced by BAPTA-AM and ionomycin in all four repeats of this experiment. However, this reduction was not statistically significant and did not correlate with the extent by which MEKK2-Lad1 interaction was compromised. Therefore, we concluded that these small changes in phosphorylation do not lead to the changes in interaction. On the other hand, the slight reduction in Tyr phosphorylation might be the consequence of this lack of association that is normally required for a proper phosphorylation downstream of c-Src (Fig. 5B), which we found to be a mediator of the EGF-induced ERK5 activation in our system (data not shown).

We next examined the Tyr phosphorylation of MEKK2 by immunoprecipitating the endogenous protein from treated HeLa cells that were transfected with either GFP-Lad1 or GFP alone. As expected, EGF stimulation of the control GFP-transfected HeLa cells induced a strong Tyr phosphorylation of the endogenous MEKK2, and this was inhibited by the c-Src inhibitor PP2 (Fig. 5C). Unlike the effect of overexpressed Lad1 on ERK5 phosphorylation, excess of GFP-Lad1 did not significantly change this effect, indicating that the Tyr phosphorylation of MEKK2 is not directly involved in ERK5 activation. Interestingly, modification of calcium concentration slightly reduced the MEKK2 Tyr phosphorylation (Fig. 5D), suggesting that this phosphorylation may be dependent in part on the calcium-dependent association of MEKK2 with Lad1.

### Changes in calcium concentrations inhibit the nuclear translocation of MEKK2

**Figure 6:**
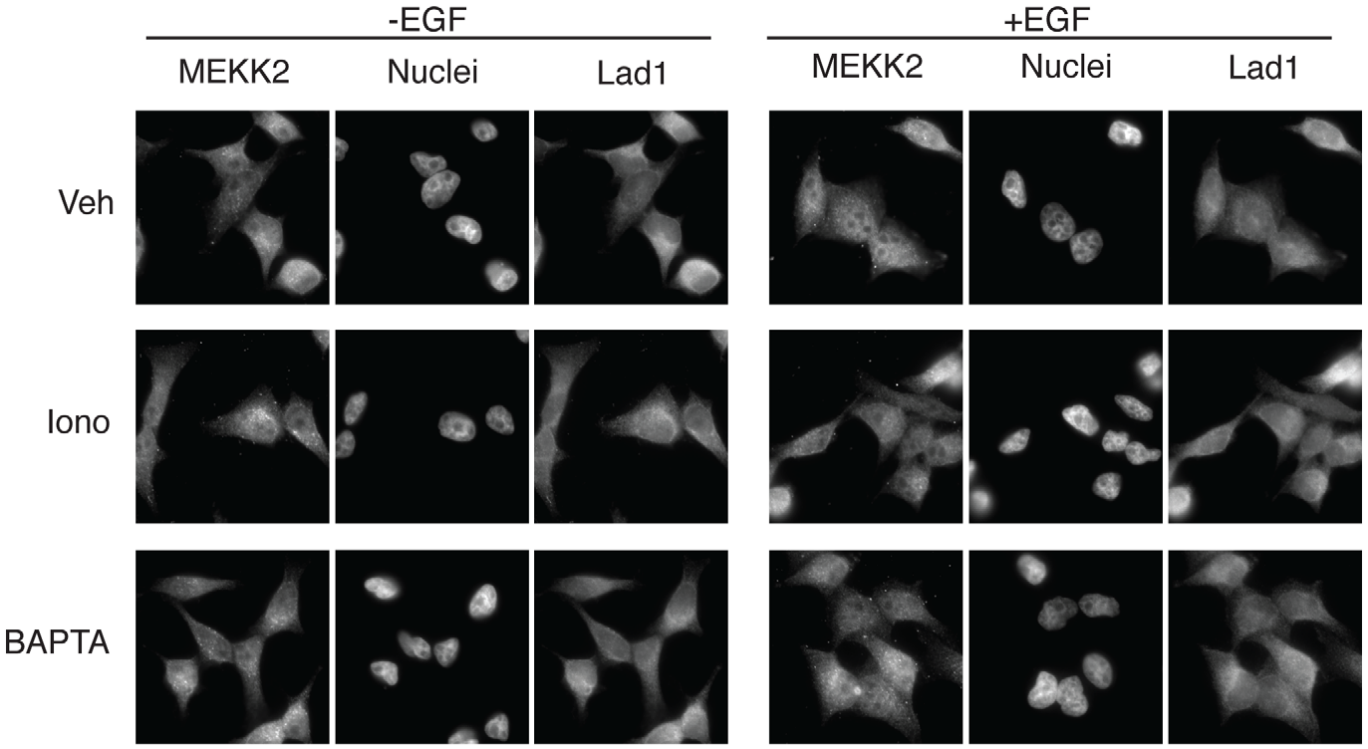
Changes in calcium concentrations do not affect Lad1 localization but inhibit nuclear MEKK2 accumulation. Serum starved HeLa cells were pretreated with vehicle, ionomycin (1 µM) or BAPTA-AM (15 µM) for 15 min and then stimulated with EGF (20 ng/ml) for 10 min. The cells were stained with anti Lad1 or anti MEKK2 Abs and visualized with fluorescent microscopy. This experiment was reproduced 3 times.

We have previously shown that ERK5 and MEK5 are localized to the nuclei of resting, as well as EGF-stimulated cells, whereas MEKK2 is distributed all over resting cells and accumulates in the nucleus upon EGF stimulation [54]. Lad1 on the other hand is localized mainly in the cytoplasm where it likely to interact with a variety of proteins and may redistribute to the plasma membrane upon stimulation [53]. To examine the effects of changes in calcium concentrations on the subcellular distribution of Lad1 and MEKK2 we immunostained HeLa cells with the proper Abs. We found that MEKK2 was indeed localized all over the cell before stimulation and accumulated in the nucleus upon EGF treatment (Fig. 6). Lad1 on the other hand was mainly localized in the cytoplasm, and its distribution was not significantly changed upon the treatment. Treatment of the HeLa cells with ionomycin or with BAPTA-AM did not significantly modify these localizations, and did not modify the cytoplasmic distribution of Lad1 upon EGF stimulation. Calcium modifiers prevented the nuclear translocation of MEKK2 upon EGF stimulation, indicating that calcium is involved in this mechanism. Thus, although the modified calcium concentrations release more MEKK2 molecules by preventing their interaction with the cytoplasmic Lad1, the free molecules do not shuttle to the nucleus, possibly because of the calcium effects on nuclear pore proteins [55]. ERK5 and MEK5 did not exhibit any change in their nuclear localization in these cells. Therefore, changes in calcium concentration influence ERK5 activity not only by modulation of MEKK2-Lad1 interaction but also by their effects on the subcellular localization of these components, possibly by inducing interactions with another set of anchoring proteins.

## Discussion

The ERK5 cascade is a MAPK pathway that plays a role in the regulation of various cellular processes. However, the mechanism of activation and regulation of the ERK5 cascade are not well understood. One signaling mechanism that was shown to regulate the MAPK cascades is the elevation of calcium levels upon stimulation [37], [38]. Indeed, we have recently found that the interaction of ERK1/2 with many proteins is regulated by calcium and dysregulation of these interactions by modulating calcium concentration leads to changes in their subcellular localization [56]. Changes in calcium can also induce ERK1/2 as well as JNK and p38 activation [42], [57]. Interestingly, in a screen aimed to identify upstream regulators of ERK5 upon EGF stimulation, we found that the most pronounced effects on ERK5 activation were achieved by inducible changes in intracellular levels of calcium. Intriguingly, we found that both increase and depletion of intracellular calcium resulted in a significant inhibition of EGF-induced ERK5 phosphorylation in EGF stimulated cells.

In order to identify the tier(s) in the ERK5 cascade that is directly responsive to the changes in calcium concentration we examined the effects of calcium modifiers on each of the cascade components. Our results clearly show that ERK5 phosphorylation by MEK5 is not affected by the calcium changes in vitro (Fig. 2). On the other hand, calcium did affect the activity of immunoprecipitated MEKK2, which may suggest that the effect is derived either from a direct influence on MEKK2 activity or from effects at upstream levels. Since it is unlikely that calcium still remains bound to the immunoprecipitated protein, we assumed that the more likely calcium target would be an upstream component. In order to identify this calcium targets, we then undertook to identify the cascade that leads to MEKK2 activation upon EGF treatment in HeLa cells. Using specific inhibitors, shRNA construct, and protein overexpression, we found that c-Src and Lad1, but not CaMKII or WNK1, participate in the process of activation of the ERK5 activation. We also confirmed previous reports [23], [53] that Lad1 and MEKK2 are Tyr-phosphorylated upon EGF stimulation downstream of the active c-Src (Fig. 5). Therefore, in our system, the pathway leading from the activated EGFR to MEKK2 activation is likely to include activation of c-Src, which then phosphorylates Lad1, which in turn associates with MEKK2. The association of Lad1 with MEKK2 allows the activation of MEKK2 through a process that may require Tyr phosphorylation of the latter, and this activation is further transmitted through the rest of the ERK5 cascade.

After elucidating the signaling components upstream the ERK5 cascade, we examined which one of them may be responsible for mediating the reduced ERK5 phosphorylation upon changes in calcium concentrations. Our results show that a strong effect of varied calcium concentration was seen in MEKK2-Lad1 interaction (Fig. 4), suggesting that this is the main process affected by the modulation of calcium concentration. On the other hand, the c-Src-mediated tyrosine phosphorylation of Lad1 was not significantly affected (Fig. 5), indicating that neither the activation nor activity of c-Src is significantly modified. The fact that the Tyr phosphorylation of MEKK2 was somewhat reduced by the calcium changes under the same conditions (Fig. 5D), may indicate that MEKK2-Lad1 interaction plays a role in allowing proper c-Src phosphorylation of MEKK2. Alternatively, it is also possible that another calcium sensitive protein kinase is mediating the c-Src-mediated MEKK2 phosphorylation. However, such an alternative would be less likely, since a Tyr-kinase cascade downstream of c-Src has not been demonstrated in any system.

The reduced MEKK2-Lad1 interaction upon pretreatment with BAPTA-AM and ionomycin (Fig. 4A) indicates that restricted and specific calcium concentrations are responsible for the interaction. This raises the question as to how this calcium effect is regulated. We found that an optimal calcium concentration is required for the MEKK2-Lad1 interaction in an in vitro assay as well (Fig. 4B), indicating that calcium influences the MEKK2-Lad1 directly, and not via any scaffold protein. We have previously shown that calcium may regulate the protein-protein interaction of ERK1/2 [40], [56], which may be mediated through the cytosolic retention sequence (CRS)/common docking motif (CD) of these ERKs. The fact that this motif contains three acidic amino acids raises the possibility that these residues function by chelating the calcium, which then add positive charges to the domain to strengthen its binding affinity. It is possible that a sequence in MEKK2 or Lad1 functions similarly, and allows their proper interaction. Indeed, MEKK2 contains several acidic and hydrophobic amino acids in residues 241**–**260 that were shown to be important for the MEKK2-Lad1 interaction [23], and these residues may function similarly to the CRS/CD domain of ERK1/2. Similar calcium effects could influence the MEKK2-MEK5 binding via the PB1 domain in the N terminus of MEKK2 [26], but, as explained above, it is unlikely that the effects of calcium on ERK5 activity are mediated through that level. In addition, it should be noted that the possible calcium chelation by acidic amino acid residues might explain the decreased interaction upon reduced but not elevated calcium concentrations. This is mainly because the limited number of acidic amino acids should restrict the number of bound calcium ions per molecule. It is possible that this higher calcium concentration causes non-specific binding of calcium, and therefore alters the net charge of the MEKK2-Lad1 proteins, but this should be further studied.

Another parameter examined here was the effect of calcium on the subcellular localization of the components of the ERK5 cascade. In a previous paper [54], we found that most ERK5 and MEK5 molecules are localized in the nucleus of Rat1 and HeLa cells. We also showed that the nuclear MEK5 is activated by MEKK2, which translocates into the nucleus upon EGF stimulation. Interestingly, other reports claimed that although ERK5 is localized in the nucleus of some cell types, it is found primarily in the cytoplasm of others [58], [59]. In the cells where the ERK5 is localized mainly in the nucleus, it is activated due to nuclear translocation of MEKK2 [54]. However, the small amount of ERK5 molecules, which are retained in the cytoplasm of these cells, are activated in the cytoplasm and then translocate to the nucleus to increase the amount of active ERK5 at this location [59].

We found that both BAPTA-AM and ionomycin inhibited the nuclear translocation of MEKK2 upon EGF stimulation. These results were somewhat surprising since we expected that the prevention of interaction with the cytoplasmic Lad1 would release MEKK2 for a faster nuclear translocation. The fact that the translocation was inhibited might indicate either that the MEKK2-Lad1 interaction is important for the nuclear translocation of MEKK2, or that calcium plays a role in the nuclear penetration of this molecule. Since we found that calcium regulates ERK1/2 translocation [56], the second possibility is more likely to occur, indicating that proper calcium concentrations are required, not only for ERK1/2, but also for MEKK2 translocation. In addition, due to the lack of correlation between Lad1 and MEKK2 phosphorylation, as well as the inhibited translocation of MEKK2 that do not correlate with the reduced binding to Lad1, it can be argued that MEKK2 might be phosphorylated and translocated to the nucleus independently of Lad1. However, our results indicate that most MEKK2 molecules do bind to Lad1 shortly after stimulation and therefore, we believe that Lad1 is essential for MEKK2 activation, which is dependent on optimal calcium concentrations. Our results also show that calcium influences the ERK5 cascade by modulating the nuclear translocation of MEKK2 as well.

In summary, this work presents novel regulatory aspects of EGF-induced ERK5 activation, mainly in regard to the role of intracellular calcium, as well as the process of nuclear translocation that accompanies this activation. We found that ERK5 activation by EGF is inhibited by both reduction and elevation of intracellular calcium levels. The changes in calcium were found to influence mainly the MEKK2-Lad1 interaction, and in vitro binding assays revealed that the proper calcium concentration is required for a direct MEKK2-Lad1 binding. The binding of these proteins is not affected by c-Src mediated phosphorylation on Lad1, but by itself slightly affects the Tyr phosphorylation of MEKK2, indicating a requirement for proper interaction to induce Tyr phosphorylation of MEKK2. In addition, we found that MEKK2 is distributed all over resting cells, and Lad1 appears mainly in the cytoplasm. Upon EGF stimulation MEKK2 accumulates in the nucleus where it can phosphorylate MEK5, while the localization of Lad1 remains unaffected. As changes in calcium levels inhibit EGF-induced MEKK2 translocation and nuclear accumulation, it is likely that this effect is due to a direct influence on its mechanism of translocation, independent of Lad1. Taken together, these findings suggest that calcium is required for EGF-induced ERK5 activation by securing the proper interaction of the MEKK2 at the MAP3K level of this cascade with the upstream adaptor protein Lad1.
